# Supplementary material for: Estimating neural activity from visual areas using functionally defined EEG templates
Source: Hum Brain Mapp. 2023 Jan 18;44(5):1846–61. doi: 10.1002/hbm.26188 (PMC9980892; doi:10.1002/hbm.26188)
Supplement: Supplementary file 1 — Figure S1. Simulation of V1 and hMT+ activity over time (in black) and the retrieved signal (in green) from EEG scalp responses using various source localization methods. Figure S2. Illustration of individual differences in EEG scalp responses. 2D (top) and 3D (bottom) representation of the scalp activity for V1 (A) and hMT+ (B) in the right hemisphere for three different individuals. Figure S3. EEG templates for a standard EGI‐system with 32, 64, 128, 256 electrodes and for a standard 10–05 system with 346 electrodes. The intensity of the color indicates the amplitude of positive (red) and negative (blue) activity. Figure S4. Crosstalk (leakage) between ROIs for different source localization methods. The amount of crosstalk (normalized for each ROI; per row) was calculated for an EEG signal simulated with an SNR of 10 and averaged across 50 individuals and 30 simulations. The darker the square, the more crosstalk between those two areas. Figure S5. The variability in EEG templates (% error) is inversely proportional to the number of participants. In log–log plots, this exponential decay function follows a 1/N slope with the intercept determined by the mean sample at N = 1. The shaded area represents 95% confidence interval. Figure S6. Simulation of ERPs with different levels of SNR for three individuals (a) and the average ERP of 20 individuals (b) at two electrodes location (Oz and Pz). Note the variation across participants and electrodes in (a). Typical recorded data will be in the 10–200 SNR range when averaged across participant. Figure S7. Source localization performance using different methods and different EEG montages with 32, 64, 128 and 256 electrodes. Brain sources are recovered from the simulation of two bilateral ROIs chosen randomly. Each datapoint represent the mean of 30 simulations with the error bars representing standard deviation. Figure S8. Source localization performance of the template method for recovering a unilateral ERP (left or right he [file HBM-44-1846-s001.pdf]

Supplementary material

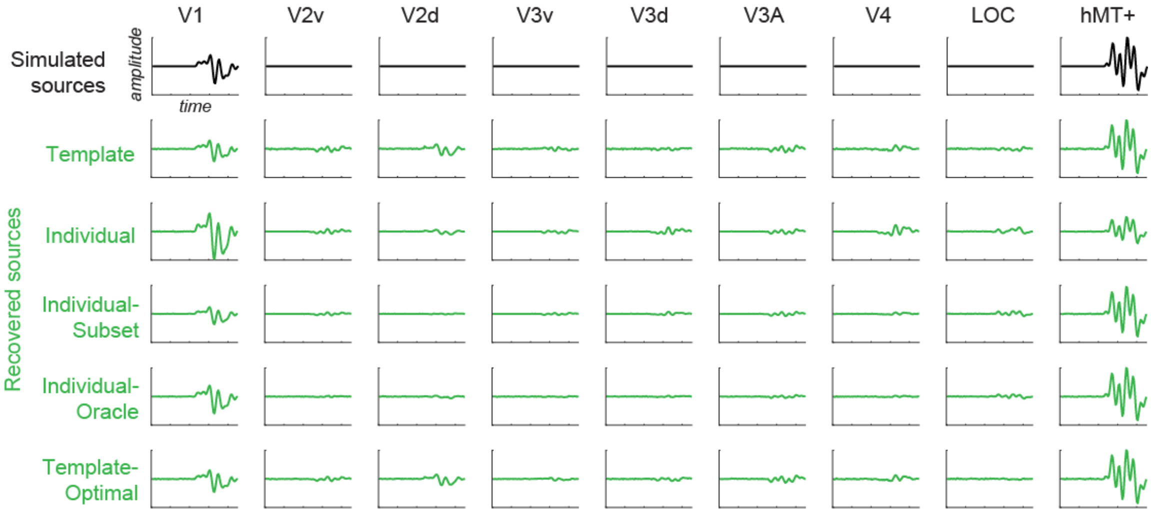

Figure S1. Simulation of V1 and hMT+ activity over time (in black) and the retrieved signal (in green) from EEG scalp responses using various source localization methods.

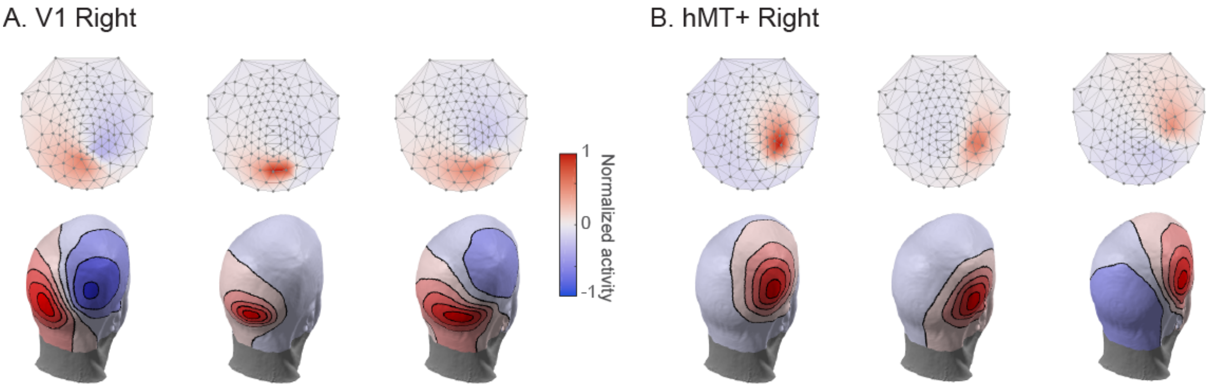

Figure S2. Illustration of individual differences in EEG scalp responses. 2D (top) and 3D (bottom) representation of the scalp activity for V1 (A) and hMT+ (B) in the right hemisphere for three different individuals.

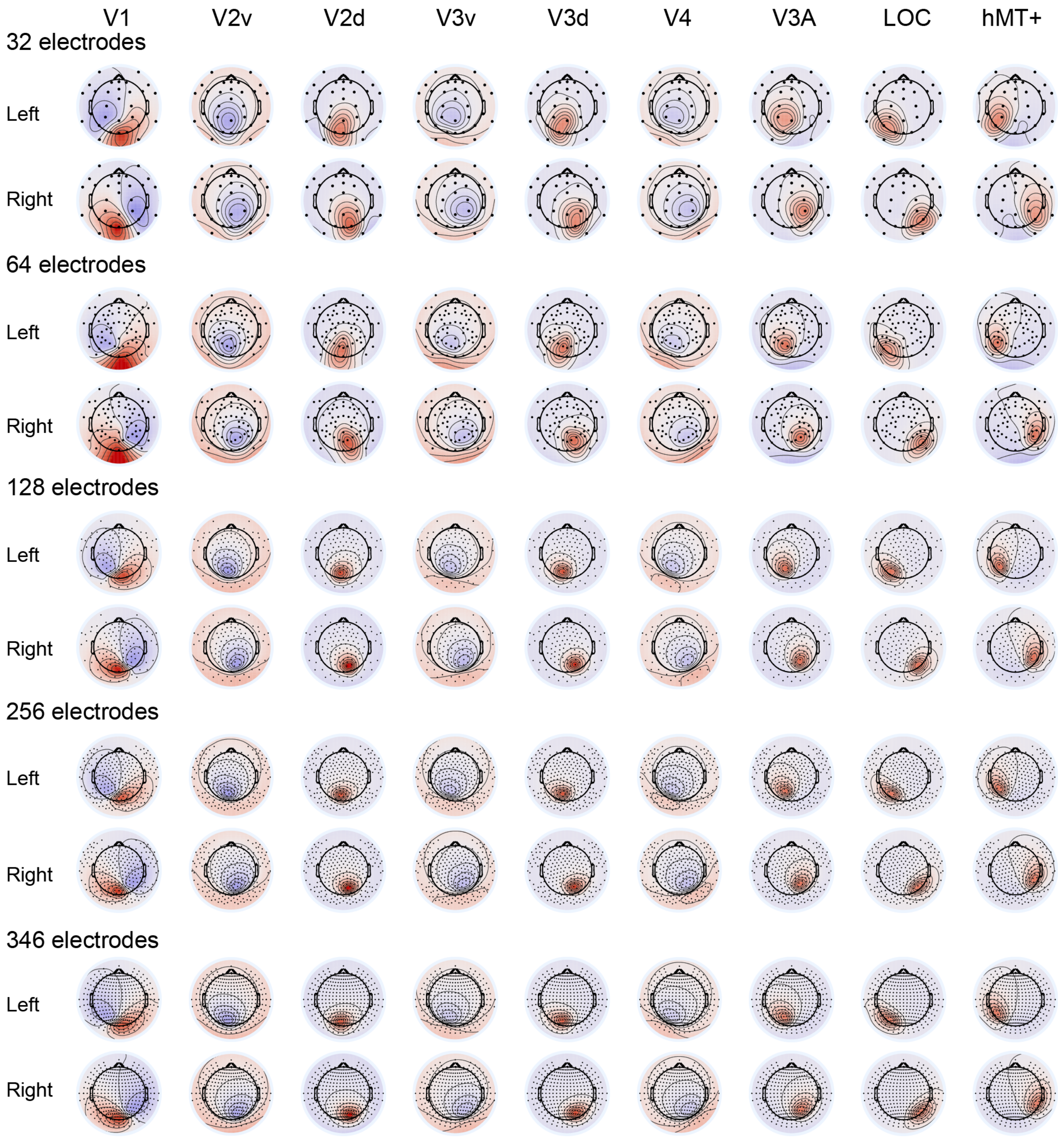

Figure S3. EEG templates for a standard EGI-system with 32, 64, 128, 256 electrodes and for a standard 10-05 system with 346 electrodes. The intensity of the color indicates the amplitude of positive (red) and negative (blue) activity.

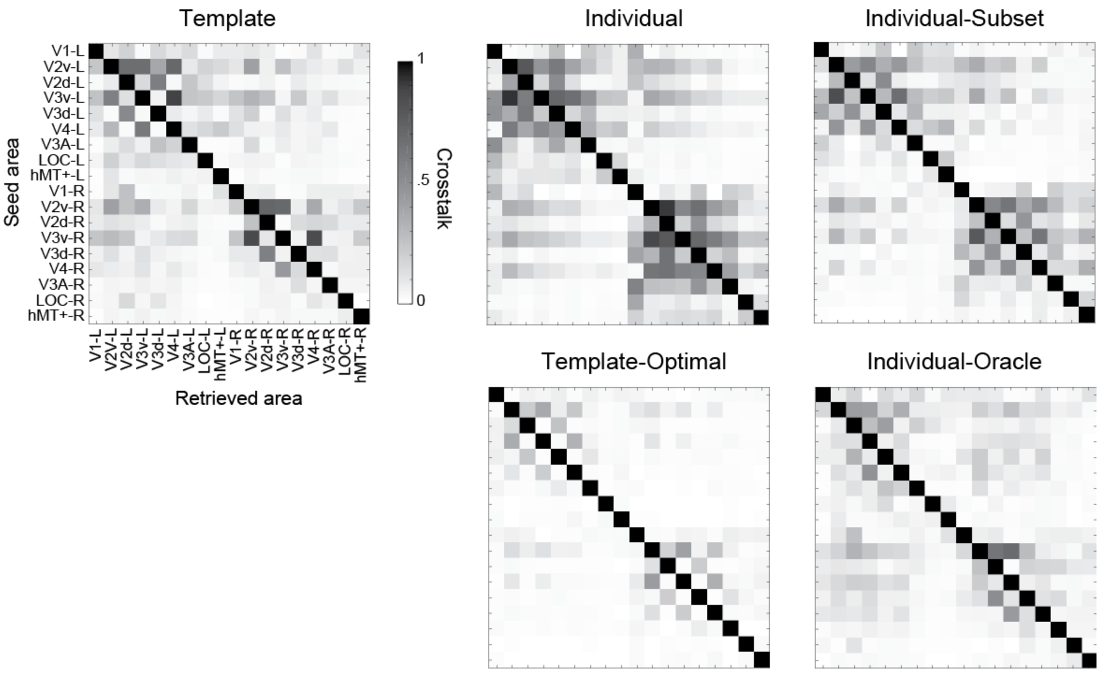

Figure S4. Crosstalk (leakage) between ROIs for different source localization methods. The amount of crosstalk (normalized for each ROI; per row) was calculated for an EEG signal simulated with an SNR of 10 and averaged across 50 individuals and 30 simulations. The darker the square, the more crosstalk between those two areas.

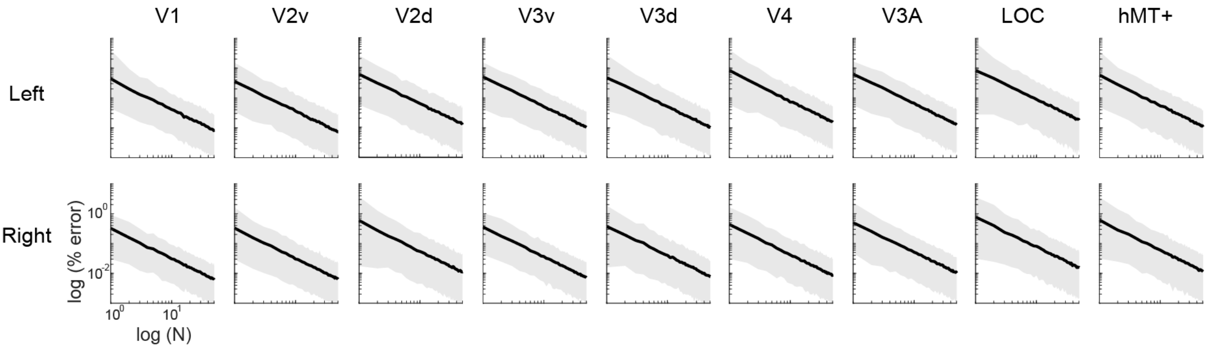

Figure S5. The variability in EEG templates (% error) is inversely proportional to the number of participants. In log-log plots, this exponential decay function follows a 1/N slope with the intercept determined by the mean sample at N=1. The shaded area represents 95% confidence interval.

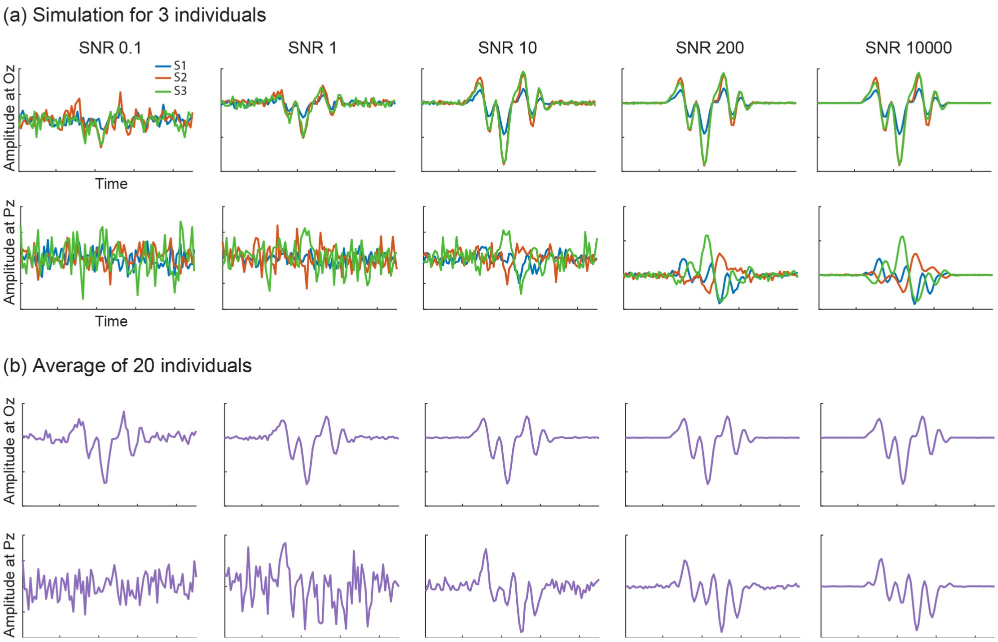

Figure S6. Simulation of ERPs with different levels of SNR for three individuals (a) and the average ERP of 20 individuals (b) at two electrodes location (Oz and Pz). Note the variation across participants and electrodes in (a). Typical recorded data will be in the 10-200 SNR range when averaged across participant.

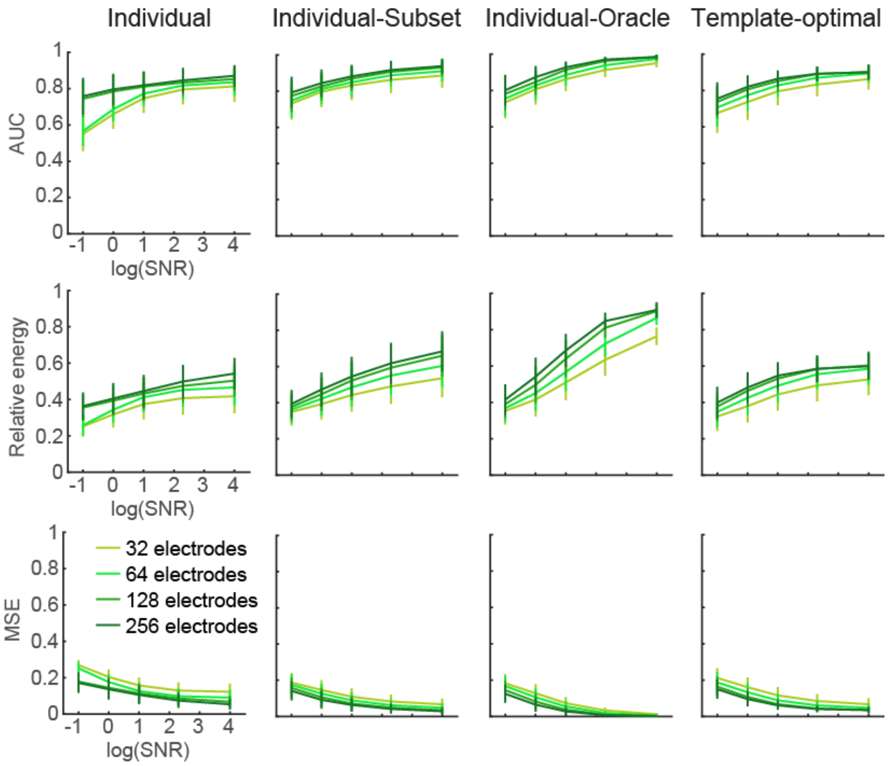

Figure S7. Source localization performance using different methods and different EEG montages with 32, 64, 128 and 256 electrodes. Brain sources are recovered from the simulation of two bilateral ROIs chosen randomly. Each datapoint represent the mean of 30 simulations with the error bars representing standard deviation.

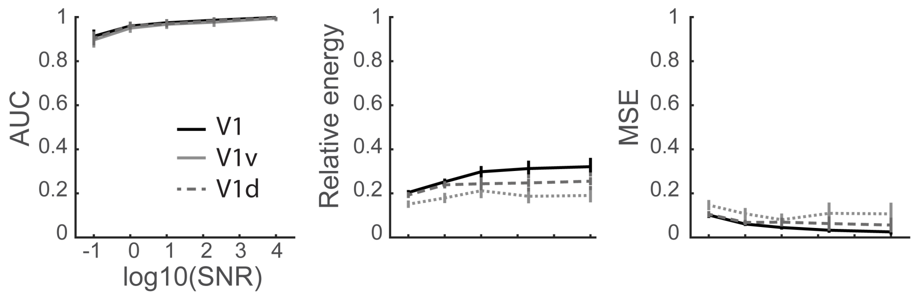

Figure S8. Source localization performance of the template method for recovering a unilateral ERP (left or right hemisphere) simulated in V1, V1 ventral (V1v) or V1 dorsal (V1d). Each datapoint represent the mean of 30 simulations with the error bars representing standard deviation.

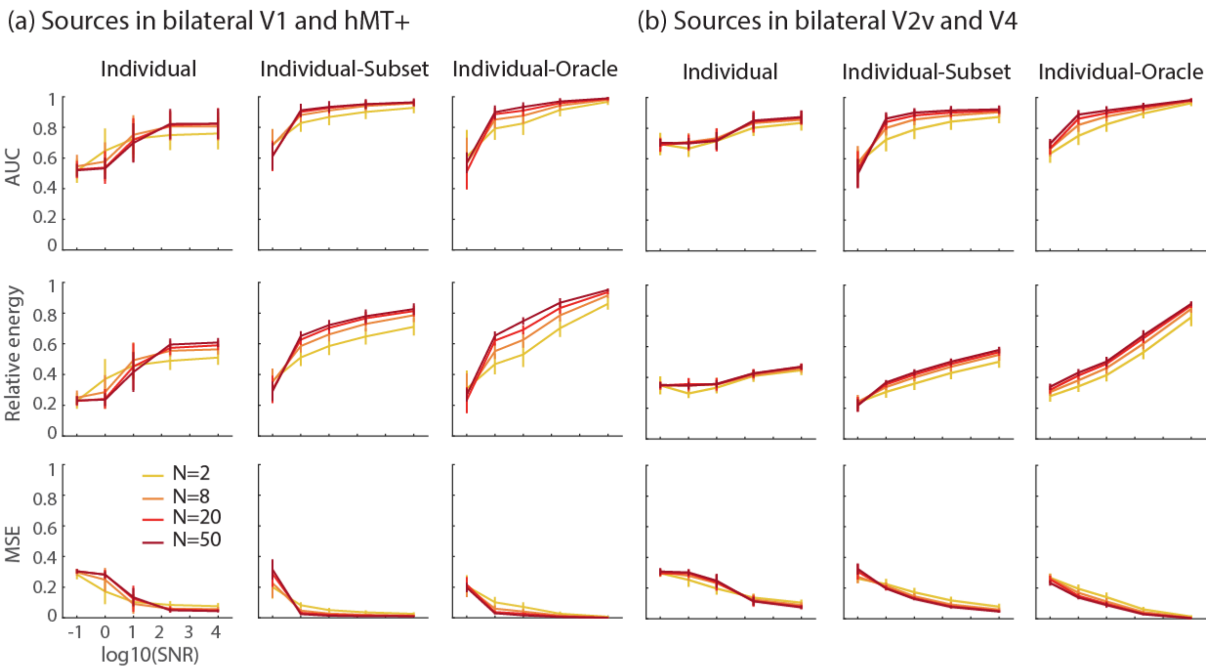

Figure S9. Source localization performance at different SNR levels for N=2, 8, 20 or 50 participants using different source localization methods with L-curve regularization. Sources are simulated bilaterally in V1 and hMT+ (a) or V2v and V4 (b). Each datapoint represent the mean of 30 simulations with the error bars representing standard deviation.

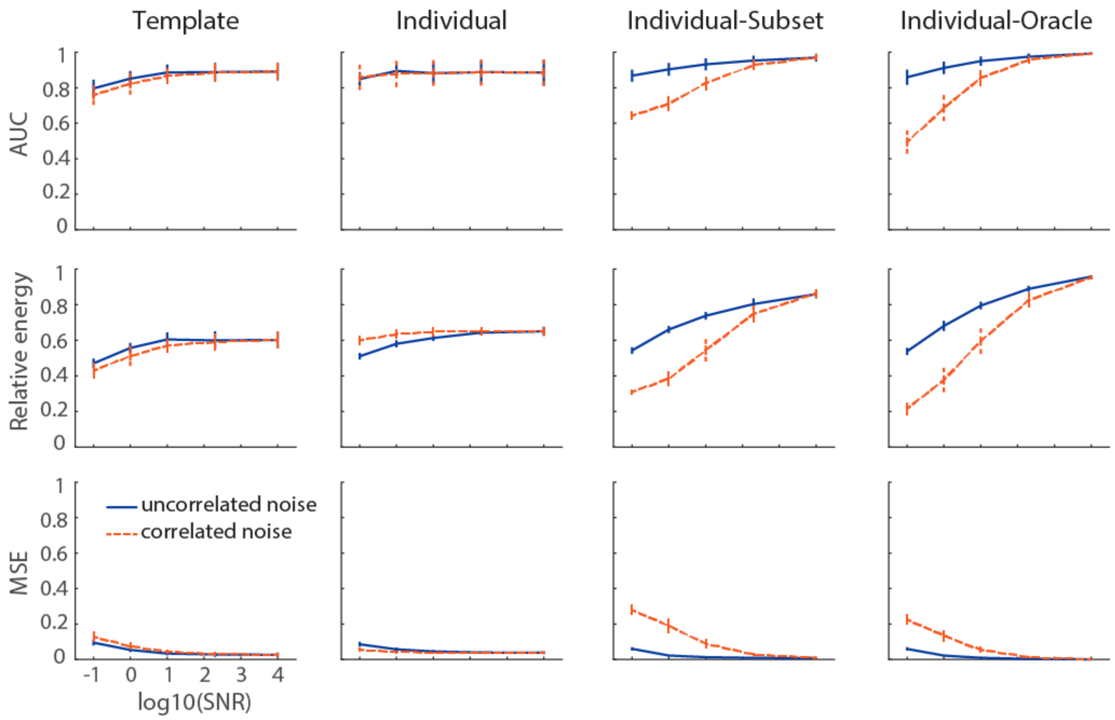

Figure S10. Source localization performance for sources simulated bilaterally in V1 and hMT+ for 50 participants with an SNR level of 10. The simulated noise was either uncorrelated (blue line) or correlated (red dashed line) across EEG electrodes at the individual level. Each datapoint represent the mean of 30 simulations with the error bars representing standard deviation. Correlated noise was created based on each participant forward model. Note that for correlated noise, the SNR level depends on each participant and on which ROI is active. Here we roughly approximate SNR levels using the same equivalence across participants. The results are almost identical for the template method with both types of noise. Performance is lower when using correlated noise for the individual-subset and individual-oracle methods with increasing level of noise. This might be due to these two methods fitting the correlated noise to non-active visual ROIs. With the individual method, the correlated noise can be fitted to visual and non-visual ROIs and therefore has little effect on the source localization performance.
